# Developing an Applied Biostatistical Sciences (ABS) network

**DOI:** 10.1017/cts.2020.506

**Published:** 2020-07-06

**Authors:** Shokoufeh Khalatbari, Dianne Jazdzyk, Janine Capsouras, Brad Downey, Eli Samuels, Cathie Spino

**Affiliations:** 1 The Michigan Institute for Clinical and Health Research, University of Michigan, Ann Arbor, MI, USA; 2 Department of Biostatistics, University of Michigan, Ann Arbor, MI, USA

**Keywords:** Applied Biostatistical Sciences Network, translational research, Michigan Institute for Clinical and Health Research (MICHR), collaboration, consultation

## Abstract

**Introduction:**

Access to qualified biostatisticians to provide input on research design and statistical considerations is critical for high-quality clinical and translational research. At diverse health science institutions, like the University of Michigan (U-M), biostatistical collaborators are scattered across the campus. This model can isolate applied statisticians, analysts, and epidemiologists from each other, which may negatively affect their career development and job satisfaction, and inhibits access to optimal biostatistical support for researchers. Furthermore, in the era of modern, complex translational research, it is imperative to elevate biostatistical expertise by offering innovative training.

**Methods:**

The Michigan Institute for Clinical and Health Research established an Applied Biostatistical Sciences (ABS) network that is a campus-wide community of staff and faculty statisticians, epidemiologists, data scientists, and researchers, with the intention of supporting both researchers and biostatisticians, while promoting high-quality clinical and translational research.

**Results:**

Since its inception in early 2018, the ABS Network has grown to several hundred faculty and staff members across a range of health and research disciplines. The ABS Network offers free trainings on innovative methods and tools in the biostatistical field, a web-based portal with resources and training lectures, and connections to U-M faculty and/or staff members for consultation and collaboration.

**Conclusions:**

Although challenging, if approached strategically, the creation of a collaboration network of biostatisticians can be accomplished. Furthermore, the process can be adopted and implemented for establishing collaboration with any network of professionals with common interests across different disciplines and professional fields regardless of size.

## Introduction

Biostatistical methods are necessary for rigorous clinical and translational research [1]. For this reason, knowledge of and access to qualified biostatisticians, who can provide input on research design and statistical considerations, is highly valued within health research universities [2,3]. Research exists on the importance and the strategies of developing biostatistical collaborations in diverse academic centers but lacks the information on process and practical approaches to establish such a network [4].

At the University of Michigan (U-M), biostatistical collaborators are drawn from non-medical school departments such as biostatistics and statistics departments, which offer a fertile and collegial environment for statisticians with a major emphasis on methods development. In addition, biostatisticians are found in health sciences schools (e.g., medicine and nursing) and within individual departments. This model often isolates applied statisticians, analysts, data scientists, and epidemiologists from each other, which may complicate their career development and job satisfaction. As with any diverse academic medical research center, establishing a community of statisticians that can effectively interact, learn, and collaborate with each other and with health research investigators is essential for optimizing the research enterprise of the U-M.

The main goals of our work were to reduce isolation of statisticians, promote the career development of statisticians through ongoing statistical training, and to improve the quality of health and health research by facilitating connections between researchers and statistical resources available across the university. We evaluated these outcomes by tracking network members’ engagement in our training and events, as well as by measuring their awareness and access of the university’s many statistical resources.

## Methods

The Michigan Institute for Clinical and Health Research (MICHR) established the Applied Biostatistical Sciences (ABS) Network in 2018. Resources and training opportunities were developed and disseminated through the network in order to catalyze a community of biostatisticians, providing networking opportunities and subsequently elevating the statistical expertise and knowledge among statisticians and researchers. The ABS network was designed to include faculty and staff from the diverse set of schools, colleges, and research units represented within the university.

**Fig. 1.**
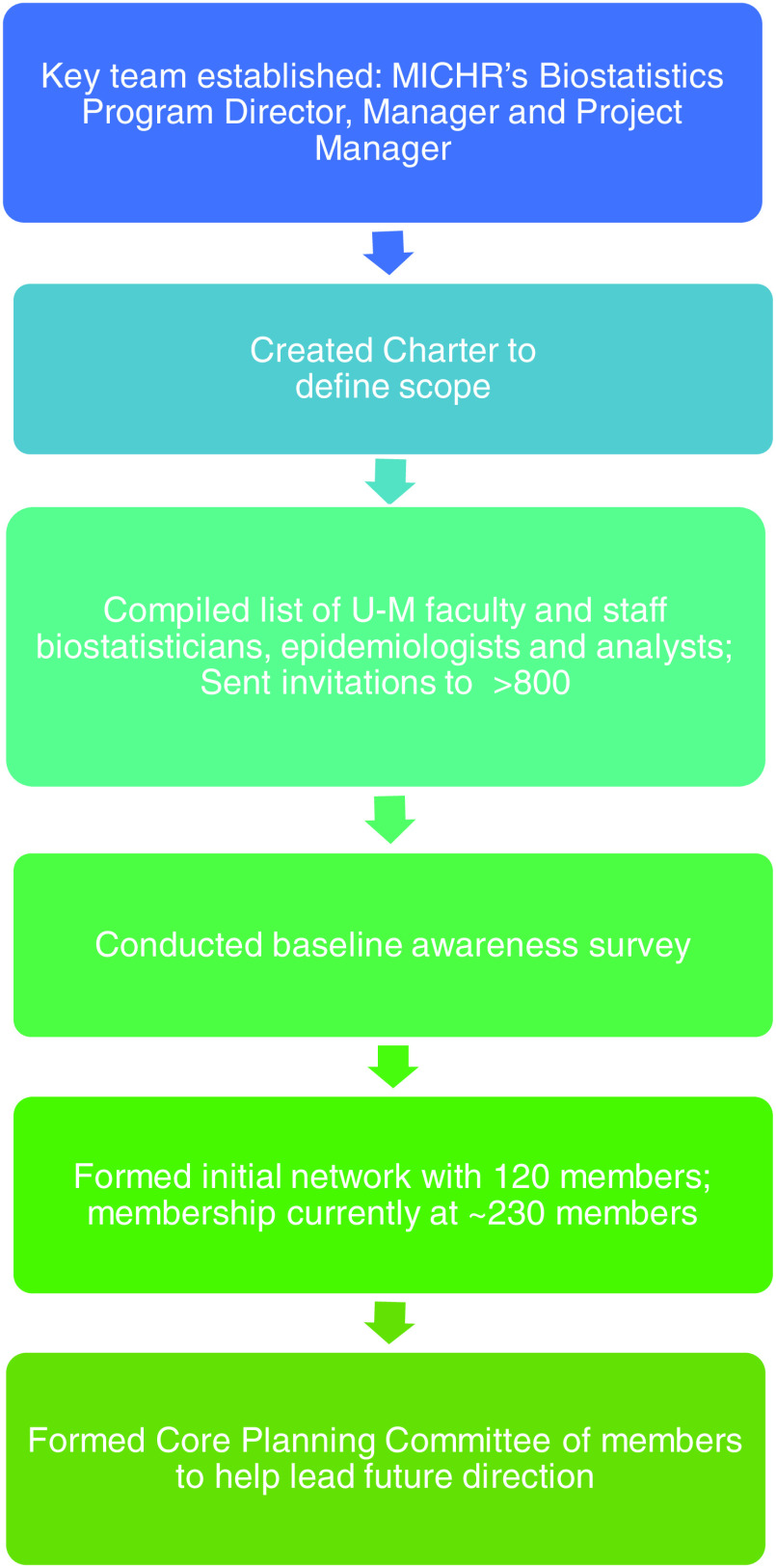
Steps taken by the project team to establish the Applied Biostatistical Sciences (ABS) Network.

Starting in 2017, several steps were taken to develop the ABS network, as shown in Fig. 1. First, a planning team was formed to lead the effort. This planning team included the Director of the MICHR Biostatistics program, the MICHR Biostatistics Manager, and an MICHR senior project manager. A program charter was created in collaboration with key MICHR stakeholders to define the scope of the project and identify the programmatic activity required to develop it. A strategic decision was made during this step that there would be no activity required for membership to be maintained in order to encourage voluntary participation in sponsored training and events. There were, however, specific activities required for completing some workshop series, namely, including a training series on Bayesian methods; participants had to attend all classes in the series and complete a capstone project.

The network was designed such that any health science researcher, clinicians, or staff members, regardless of biostatistical training, would be welcomed to join. In short, the network was meant to be inclusive of the university’s entire biostatistical research community.

In addition, if clinical researchers indicated they wanted to join to learn more about statistical methods and make connections with ABS members, the network was designed to include them too. However, it was also decided to exclude actively enrolled students from the network, both due to their necessarily limited biostatistical expertise, relatively short tenure at the U-M, as well as the need to focus on and facilitate ongoing participation among the network members.

The mission of the ABS Network was stated so anyone joining would see that its activities were geared towards applied biostatisticians whose primary job responsibilities include proper data collection, analysis, and reporting of the results.

A key initial step of the project required the identification of all relevant faculty and staff biostatisticians working at the university, with a particular focus placed on those involved in clinical and translational research. Staff and faculty names, email addresses, titles, and appointments were collected from multiple institutional resources including the university’s employee directory, human resource (HR) records, as well as department and unit websites.

To supplement these records, department chairs and managers of university statistical centers and interest groups on campus were contacted and asked to help identify potential ABS network members. The recruitment process was also repeated annually to enable the ongoing growth of the network.

It is important to note that it was not possible to identify all potential members for the ABS Network since university job titles for staff and faculty do not necessarily reflect individuals’ expertise or professional interests with perfect accuracy. For example, some but not all U-M staff members with a job title of Research Area Specialist perform statistical analyses as part of their professional work.

The annual survey was used to evaluate ABS Network members’ and non-members’ awareness and use of statistical resources at the university as well as how the network members’ professional development needs were being met. The survey was sent to over 830 faculty and staff members each year in an effort to reach as many potential members as possible. The survey was administered online using Qualtrics [5].

In addition to inviting respondents to join the ABS network, the survey included questions assessing awareness and use of biostatistical resources and centers at the university, such as the Center for Statistical Consultation and Research, Institute for Healthcare Policy and Innovation, Statistical Analysis of Biomedical and Educational Research Group, and the Center for Healthcare Outcomes and Policy. Respondents were also asked questions about their professional status and background including their credentials, fields of study, and job roles. This survey was administered annually, both as a recruitment mechanism and to measure change in awareness and use of statistical resources over time.

A series of meetings and workshops were organized by MICHR for all ABS members. In addition, a series of lectures were offered to the university’s entire research community, to which all ABS network members and non-members were routinely invited. Event communications were sent by email to network members, posted on MICHR’s website, promoted in relevant U-M newsletters, and posted as hard-copy announcements around the U-M medical campus. In an effort to increase interactions of members, monthly social lunch gatherings were organized. Multimodal communications were also used to promote these events as well as related resources.

To further facilitate network members’ access to statistical resources, the project team created a web-based portal (https://www.um-biostatnetwork.org/) using Squarespace® website builder and AirTable spreadsheet application to house a library of resources, a membership directory, and information about ABS Network events; these resources were all subsequently made available to all Clinical and Translational Science Awards Programs in the USA to disseminate ABS Network resources beyond the university.

For the membership directory, members complete an online form with their name, department, job title, expertise, and email addresses. A review was performed of American Statistical Association website and other statistical groups to identify types of research. These categories were finalized and decided on in collaboration with programmatic stakeholders. “Clinical research” was included as an expertise area as many of network members work in clinical research areas and not specifically in the area of statistics per se.

Paper and online forms were used to evaluate the quality and impact of each training workshop in order to measure how attendees perceived each event to be engaging, as well as relevant and useful to their individual work. In addition, these surveys solicited attendees’ suggestions for future lecture topics as well as potential logistical improvements for the events themselves.

The project team reported all survey results, network training, event and lecture attendance, and analysis of ABS Network membership background to internal stakeholders, including MICHR’s faculty leadership. In alignment with established evaluation frameworks [6], programmatic stakeholders collaborated to interpret the results of these analyses, develop a narrative explaining the trends that were observed, and finally, identifying evidence-based programmatic improvements with the potential to enhance the impact of the ABS network. This evaluative work led to the development of an innovative workshop series on Bayesian methods which was piloted through the network.

## Results

**Fig. 2.**
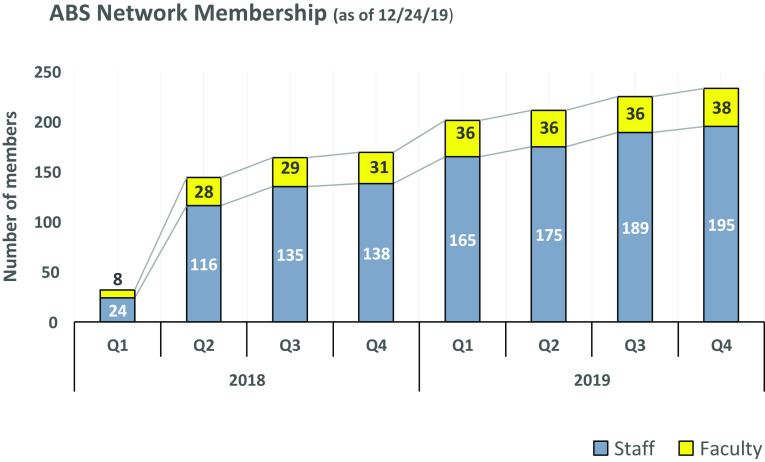
Membership numbers 2018 through 2019. ABS, Applied Biostatistical Sciences.

In the two years since its inception in 2018, the ABS Network has grown to include more than 230 members. This membership constituted about one-third of the population of potential members initially identified for recruitment. Roughly, 15% of the ABS network members held a faculty appointment. Collectively, university faculty and staff in the network worked in over 80 departments across 17 schools and colleges of the university (Fig. 2).

Survey results suggest that just under half of the network members held a degree in biostatistics (26%), statistics (12%), or epidemiology (9%). Approximately, a third of members had earned a doctorate degree. The majority of members responding to the survey identified themselves as a statistician, biostatistician, data analyst, data scientist, epidemiologist, clinician, researcher, or other type of researcher. Non-member respondents to the 2020 survey were asked why they had not volunteered to join the ABS Network. The majority (75%, or 80 individuals) indicated that they were previously unaware of the network, that they lacked the time to be a member, or that the focus of network was not applicable to their professional interests.

ABS Network members and non-members’ survey responses regarding their awareness and use of the university’s statistical resources were compared over 2 years using the results of the 2019 and 2020 annual surveys. In 2018, a total of 833 individuals received the survey, of which 60 responded; 975 individuals received the 2019 survey of which only 115 responded and of the 885 individuals surveyed in 2020 only 120 responded. The results to these surveys, with limitations of the very low response rate (7%, 12%, and 14%, respectively) notwithstanding, indicated that well over 80% of respondents were aware of at least one statistics resource at the university over this time period. There was no discernable increase in members’ awareness of statistical resources over time or meaningful difference between network members and non-members’ awareness of resources.

In contrast, there were notable increases in network members’ use of statistical resources over time and in comparison to non-members. Specifically, members’ use of university resources as well as their referral of these resources to others grew between 2019 and 2020 (69% to 78% and 48% to 65%, respectively). Moreover, these rates increased substantively in comparison to those of non-members’ (48% to 56% and 43% to 21%, respectively). While rates of resource awareness and use were higher among ABS Network members than non-members, the likelihood of this trend being a result of a selection effect was also expected as members of the network were both recruited on an ongoing basis from within the population of non-members.

Because the 2018 results generally suggest that many university statisticians were aware of university resources that they were not actively using, over the course of 2019, the project team increasingly focused on providing members training and networking opportunities directly through the network itself. Specifically, in response to these results, core planning committee comprised 12 volunteers, representing nine departments across the university was formed, to identify training opportunities and resources which were relevant to the existing membership. Following this committee’s recommendations, to further promote engagement among the ABS Network community, four quarterly events were planned for each year, including two lectures and two ABS members-only meetings (Table 1).

**Table 1.** Applied Biostatistical Sciences (ABS) network lectures/meetings for research community

Year/Quarter	Event
2018/1	Lecture: Comparing and communicating adaptive designs for a dose-ranging study
2018/2	Member-only meeting: Missing data parts I and II – concepts, approaches, and imputation methods
2018/3	Lecture: Statistics and data science communication: tips and tricks for a new model of engagement
2018/4	Member-only meetings: Managing research partnerships parts I and II – statisticians are from Mars, clinicians are from Venus
2019/1	Lecture: Biomarker-driven clinical trial designs
2019/2	Member-only meeting: Statistical tools for healthcare policy and outcomes research
2019/3	Member-only meeting: Panel discussion on the role of statisticians versus data scientists
2019/4	Lecture: Clinical trial data monitoring committees: the basics and some controversies Member-only meeting: Panel discussion: expectations of being a statistician member of Data and Safety Monitoring Boards (DSMBs) of clinical trials, best practices for statisticians who report to DSMBs, and issues and controversies that arise during the monitoring of ongoing trials
2020/1	Lecture: Sequential, multiple assignment, randomized trial clinical trial design for tailored treatment guidelines

A main goal of the ABS Network project team was reducing the isolation of biostatistical collaborators. Success was evaluated by looking at the membership, and their level of engagement by participating in our events as well as their access of the online resources provided through the ABS Network website.

General data from the website was reviewed using Google Analytics to assess user visits, session duration, and pages visited the most. In 2019, the website’s homepage was visited 813 times with overall number of page views of 2,443 times. The top three visited pages included: 236 visits to the events page, 147 visits to the lectures and meeting materials page, and 133 visits to the statistical tools and resources page.

Data derived from Google Analytics also suggest that website visits varied substantively over time but also that email promotions of ABS Network event were associated with spikes in the number of website spike that occurred shortly after email correspondence was sent. Event attendance records and post-workshop surveys indicated that network members were active and engaged participants reported receiving training was useful to their work.

On average, more than 60 statisticians and researchers attended each of the ABS network training opportunities, with 88% of post-event survey respondents indicating they found the events useful and engaging. While some of the trainings received somewhat higher ratings for their quality, relevance and utility than others, the attendees’ evaluations of the events were consistently favorable with no notable differences found between members and non-members or between research investigators or staff. The results of all relevant surveys and event evaluations were periodically shared with the program stakeholders who both interpreted the results and informed the conclusions of the project team.

Any interpretation of these findings must be made in the context of measures of the effort required to produce them. In the first three months, staff efforts to initiate creation of the network took about 35% of administrative time (administrative assistant or project manager) and 20% time for the technical lead. Over the first year, about 20% administrative time (administrative assistant or project manager), 10% of the technical lead time, and 5% faculty lead time were needed.

The implementation of the ABS Network also led to demonstrable and innovative improvements in the training opportunities utilized by some biostatisticians at the university. Most notably, in response to member interests, the project team partnered with five biostatistical faculties to provide a Bayesian analysis workshop series to introduce statisticians to Bayesian principles and analytical tools which can be applied to applied research problems. Twenty network members participated in the six monthly training sessions, which culminated in a capstone project where members analyzed datasets from one of their existing research projects using Bayesian methods. Among the 20 participants, 11 participants collaborated in four different teams for their capstone projects, one of which was submitted for publication in a peer-reviewed journal.

## Conclusion

The process for creation of the ABS network can be adopted and implemented for establishing collaboration with any network of professionals with common interests across different disciplines and professional fields regardless of size.

Efforts to establish an infrastructure that encourages effective collaboration between clinical researchers, statisticians, and other key research personnel is not unique to the U-M. Other research institutions have also recognized the importance of such fluid collaborations in promoting high-quality, productive research initiatives. For example, Koehlmoos et al. created a coordinated health services research system that provides a platform for new faculty, researchers, residents, and students to collaborate with senior researchers and receive trainings [2]. This effort was proven effective in increasing the number of publications and presentations and improving the quality of such work as well as its utility to inform policy making for the Military Health System.

The growth of the ABS network and active use of the training opportunities and resources disseminated through the network indicate that MICHR is contributing to the development of an active community of practicing biostatisticians at the U-M. The positive evaluations of the training opportunities provided through the network were likely attributable both to the collaborative selection of training topics by the project team and network members as well as to the high quality of the subject matter experts the project team was able to recruit as presenters and lecturers. The direct provision of training opportunities through the network and the collaborative identification of training topics are both potential best practices that can be replicated by other research centers forming similar networks.

There were many challenges to the development of the ABS Network which may be relevant to other institution’s effort to replicate this approach locally. Particularly in the initial search to identify applied biostatisticians or staff with a potential interest in biostatistics because many staff performing statistical work could not be easily identified by their job title. This problem was further complicated by the myriad of job titles utilized in the University’s HR records for research team members. For example, many statisticians have the title “Research Area Specialist.” In addition, many members and potential members were physically scattered across the university’s large campus, necessarily limiting their ability to participate in ABS events, particularly the social gatherings. Finally, while the collaborative approach taken to identifying training topics was considered successful, selecting topics of interest for events and the implementation remained administratively burdensome.

These lessons learned and positive results of the ABS Network’s implementation at the U-M have informed the identification of the next steps for the project team and MICHR. The team will endeavor to ensure that training in cutting-edge methods and tools offered through the ABS Network continue to be matched the emergent needs and interests of the networks’ growing membership.

The evaluation of the impact of the network must also extend beyond measures of members’ awareness and utilization of statistical resources at the university and be conducted in ways that better enable the rigorous analysis of changes in member behavior. For this reason, MICHR aims to conduct longitudinal, identifiable surveys in order to track ABS network members’ long-term engagement with different statistical units across campus, including not only their referrals to colleagues and their direct utilization, but also their informal networking, consultations, access of statistical resources, and development of study partnerships.

In addition, the project team aims to more actively promote career development opportunities through the network, particularly those relevant to early career statisticians who are not housed in a department with formal structures for professional development. The team also plans to further facilitate collaboration between clinicians and network members by connecting researchers at U-M who are seeking biostatistical collaborators. As a result of these next steps, it is expected that the ABS network will facilitate the development, formation, and sustainability of research teams using cutting-edge biostatistical methods to advance clinical and translational science [1,7].
